# Gut homeostasis and regulatory T cell induction depend on molecular chaperone gp96 in CD11c^+^ cells

**DOI:** 10.1038/s41598-017-02415-7

**Published:** 2017-05-19

**Authors:** Yunpeng Hua, Yi Yang, Shaoli Sun, Stephen Iwanowycz, Caroline Westwater, Boris Reizis, Zihai Li, Bei Liu

**Affiliations:** 1 Department of Microbiology and Immunology, Hollings Cancer Center, Medical University of South Carolina, Charleston, South Carolina United States; 2 Department of Pathology and Laboratory Medicine, Medical University of South Carolina, Charleston, South Carolina United States; 3 Department of Oral Health Science, Medical University of South Carolina, Charleston, South Carolina United States; 4 Department of Pathology and Medicine, Langone Medical Center, New York University, New York, United States; 5 Department of Hepatobiliary Surgery, Sun Yat-sen University, Guangzhou, Guangdong Province China

## Abstract

The intestinal immunity and tolerance are orchestrated by both the innate and the adaptive immune system. Intestinal professional antigen presenting cells (pAPCs) recognize and respond to the gut microbiota through multiple pattern-recognition receptors, including TLRs and NLRs. How gut pAPCs maintain mucosal homeostasis remains incompletely understood. Heat shock protein gp96, also known as grp94, is an essential immune chaperone for TLRs. However, the role of gp96 in regulating CD11c^+^ APCs in the gut immunity and tolerance is unknown. By a genetic strategy, we report here that selective deletion of gp96 from CD11c^+^ cells in mice results in alteration of dendritic cell and T cell subsets in the gut as well as loss of antigen-specific regulatory T cell induction in the mesenteric lymph nodes. Strikingly, these conditional gp96-null mice developed spontaneous colitis, had increased levels of systemic and fecal IgA, and were highly susceptible to chemical-induced colitis. Our findings for the first time demonstrate that gp96 is essential for CD11c^+^ cells to induce regulatory T cells and maintain gut homeostasis, illustrating the importance of protein immune chaperone in safeguarding against immune pathology.

## Introduction

Professional antigen presenting cells (pAPCs) play a critical role in regulating both innate and adaptive immune responses^1^. In the intestine, pAPCs including dendritic cells (DCs) and macrophages are strategically positioned to protect the gut while maintaining mucosal tolerance to food, self-antigens and microbiota. Lamina propria (LP) DCs are a heterogeneous group of cells with their subsets and functions being continuously defined^2–4^. There are two major functionally distinct subsets of DCs based on the surface expression of CD103 and CX_3_CR1^5, 6^. CD103^+^ DCs originate from the monocyte and DC progenitor (MDP) via the Flt3 ligand-dependent pathway^7^. CD103^+^ intestinal DCs consist of CD103^+^CD11b^+^ DCs controlled by the transcription factors IRF4 and Notch2^8–10^ and CD103^+^CD11b^−^ DCs that require BATF3 and IRF8 for their respective development^11^. CD103^+^ DCs preferentially promote the differentiation of Foxp3^+^ regulatory T (Treg) cells to maintain tolerance^12–14^. In addition, a recent study showed that CD103^+^CD11b^−^ DCs are required for peripheral Treg cell induction during dietary antigen exposure^2^. In contrast, CX_3_CR1^+^ DCs are derived from Ly6C^hi^ monocytes and their expansion requires GM-CSF^7^. CX_3_CR1^+^ DCs induce both Th1 and Th17 cell differentiation in the gut^6, 7, 15^. However, intestinal macrophages also express CX_3_CR1 and induce Th1 cells during colitis^16^. Moreover, a recent study showed that CX_3_CR1^+^ macrophages but not CD103^+^ DCs are essential for the generation of segmented filamentous bacteria (SFB)-specific Th17 responses^17^. The regulation of pAPCs by metabolic pathways^18^, unfolded protein response^19^ and protein chaperones^20^ is an emerging interest in the field, considering pAPCs can respond to a broad array of environmental cues, e.g., pathogens and injuries, to restore tissue homeostasis^21^. However, how pAPCs maintain mucosal homeostasis remains incompletely understood.

Despite the thick mucus layer, interaction between gut pAPCs and commensal microbiota does occur in the homeostatic setting^22–24^. pAPCs recognize and respond to microbiota through multiple pattern-recognition receptors, including Toll-like receptors (TLRs), NOD-like receptors (NLRs), RIG I-like receptors, C-type lectins and mannose receptors^25–27^. Most studies on the intestinal pAPC biology so far have taken a reductionist approach. For example, TLR2, TLR4 and MyD88 deficient mice are found to be highly susceptible to dextran sulfate sodium (DSS)-induced colitis^28–30^. Dysregulation of interactions between the gut microbiota and the mucosal immune system causes development of chronic intestinal inflammation, which is mediated by DCs through their unique role in priming T-cell responses^31^.

Heat shock protein gp96^32^, also known as grp94^33^, is a molecular chaperone and the most abundant and ubiquitous protein in the lumen of the endoplasmic reticulum (ER). gp96 is constitutively expressed in most cells and its expression is induced by ER stress triggered by the accumulation of misfolded proteins in the secretory pathway^34^. Recent genetic studies from our group and other laboratories have established gp96 as a master molecular chaperone for most TLRs^7, 35–38^. It chaperones TLRs in concert with PRAT4A (also known as CNPY3)^39^. gp96 is also an essential chaperone for multiple integrins^36–38^, platelet glycoprotein Ib-IX-V complex^40^, GARP^41^ and Wnt co-receptor LRP6^42^. Thus, protein quality control and innate immunity appear to converge molecularly on gp96. The fact that gp96 chaperones multiple innate receptors also creates an experimental opportunity for us to genetically and simultaneously examine the roles of gp96 and its client network in immune homeostasis. Our recent study demonstrated that macrophage-specific gp96-knockout mice are more resistant to DSS-induced colitis^43^. These macrophage-specific gp96-knockout mice have significantly less inflammations in the colon and lower percentages of Th17 and Th1 cells in colonic lamina propria (cLP) compared with their wild type (WT) littermates^43^, suggesting a critical role of gp96 and its clientele (such as TLRs) in myeloid cells in exacerbating intestinal inflammation. However, the roles of gp96 in CD11c^+^ pAPCs have not been examined *in vivo*. In this study, we generated a unique CD11c^+^ cell-specific gp96 knockout (KO) mouse model to address the contribution of gp96 to the biology of CD11c^+^ cells in mucosal immunity and tolerance. We report that specific deletion of gp96 in CD11c-expressing cells led to a significant alteration of DCs and T cell subsets in the gut, including a large reduction of Treg cells, and the loss of antigen-specific Treg cell induction. Strikingly, we found that CD11c^+^ cell-specific gp96 deficient mice develop spontaneous colitis with age (~24 weeks). We also found that these gp96-deficient mice are highly susceptible to DSS-induced colitis and have significantly increased systemic and fecal IgA levels. Thus, our study for the first time demonstrates the fundamental roles of gp96 and its client network in CD11c^+^ cell biology and gut tolerance.

## Results

### Generation and characterization of CD11c^+^ cell–specific gp96-deficient mice

**Figure 1:**
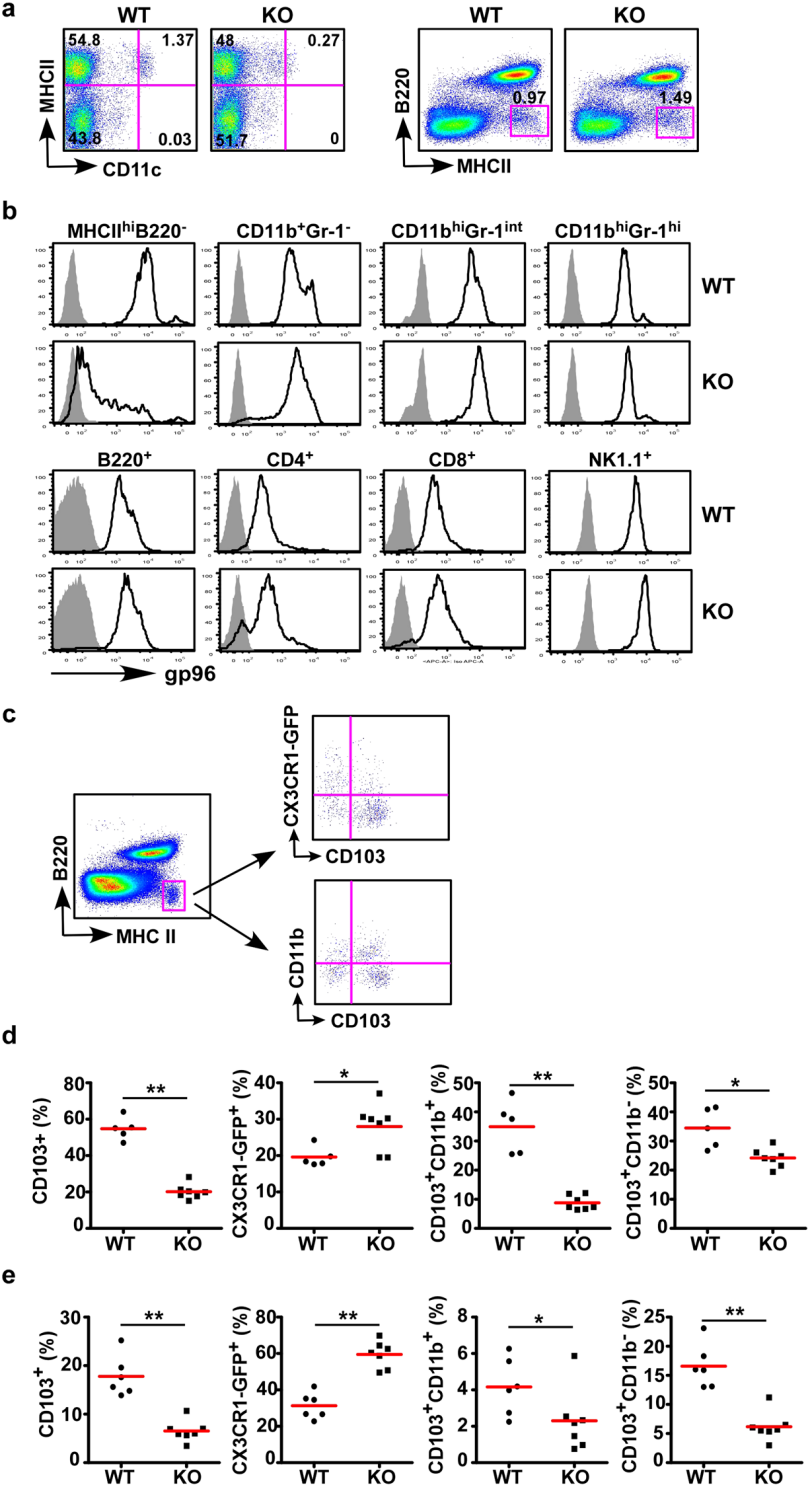
Deletion of gp96 in CD11c^+^ cells results in alteration of pAPC subsets in the gut. (**a**) Flow cytometry analysis of splenic classic DCs by gating with CD11c^hi^MHCII^+^ (left panel), or negative gating strategy to define DCs (B220^−^MHCII^+^, right panel). (**b**) Intracellular analysis of gp96 expression in different lineages of hematopoietic cells in the spleens of KO and WT mice (open histogram with solid line). Gray-shaded histograms represent isotype controls. (**c**) A representative flow cytometry analysis of pAPCs. The pAPC populations was first gated on B220^−^MHCII^+^ cells, and then further analyze different subsets of APCs in MLN and cLP based on CX_3_CR1-GFP, CD103, and CD11b expression markers. Numbers represent % of cells in each quadrant. (**d**) Quantification of CX_3_CR1^+^, CD103^+^, CD103^+^CD11b^+^, and CD103^+^CD11b^−^ subsets as defined in (C) in MLN of WT and KO mice. (**e**) Quantification of CX_3_CR1^+^, CD103^+^, CD103^+^CD11b^+^, and CD103^+^CD11b^−^ subsets as defined in (C) in cLP of WT and KO mice. Eight to 12 week old mice were used (n = 5–7). *p < 0.05; **p < 0.01.

**Figure 2:**
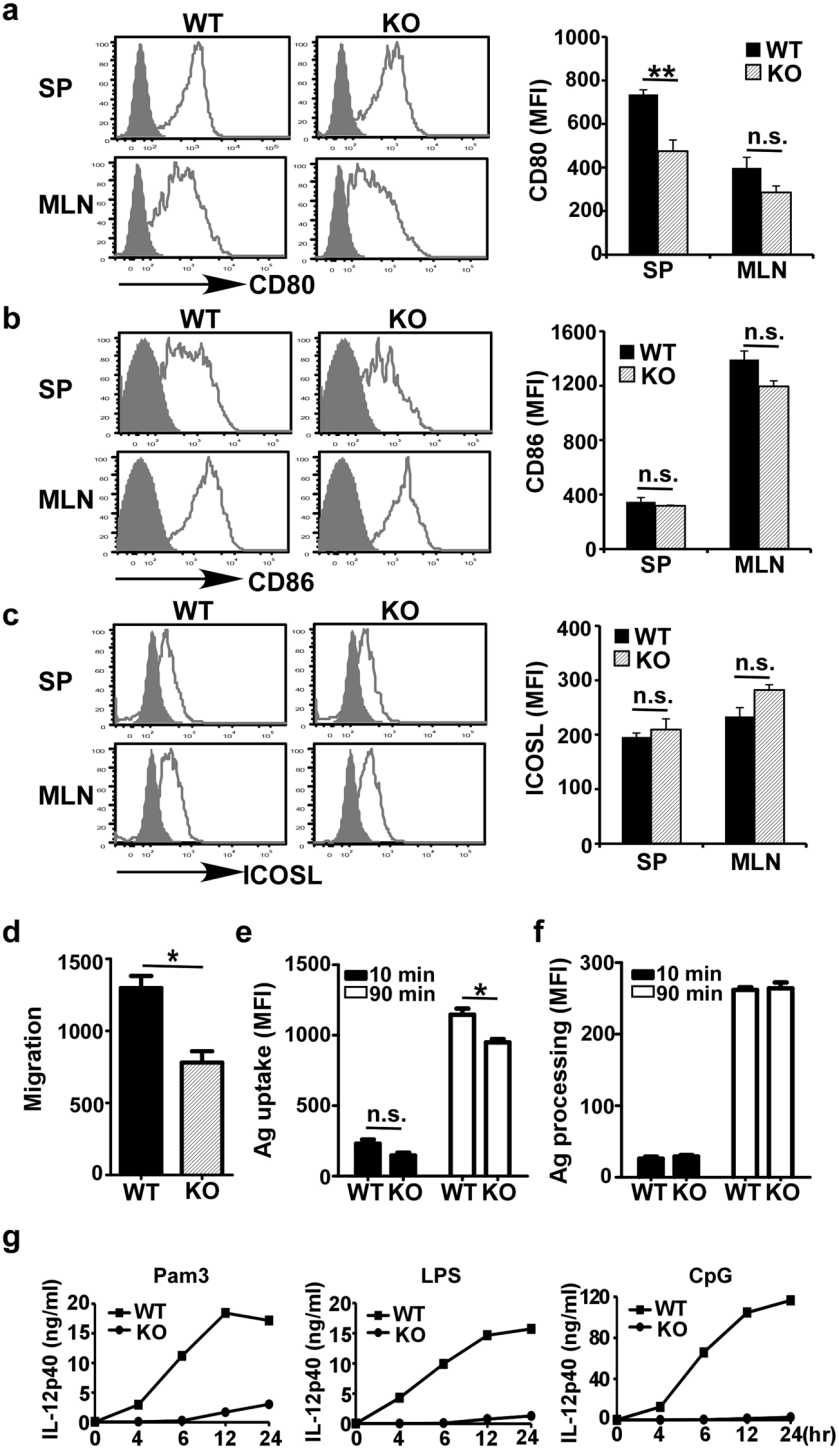
Deletion of gp96 in CD11c^+^ cells results in alteration of their migration and unresponsiveness to TLR ligands. (**a**–**c**, left) Flow cytometry analysis of CD80 expression (**a**), CD86 (**b**), ICOSL (**c**) (open histogram) in dendritic cells (DCs) from the spleen (SP) and mesenteric lymph nodes (MLN). Shaded histogram represents background stain. (**a**–**c**, right) Mean fluorescence intensities of different surface markers. (**d**) *In vitro* migration assay. Isolated splenic DCs from WT and KO mice were measured in response to CCL21. Y axis showed the number of migrated cells. (**e**,**f**) *In vitro* antigen uptake and processing. (**g**) Bone marrow derived DCs were incubated with Pam3, LPS, and CpG for different time points. Supernatant was harvested and IL-12p40 was measured by ELISA. Error bars indicate SEM (n = 3). *p < 0.05; **p < 0.01; n.s.: not significant.

pAPCs including DCs and macrophages play a critical role in both innate and adaptive immune responses. To study the role of gp96 in pAPC function, we generated CD11c^+^ cell-specific gp96-deficient mice by crossing our *Hsp90b1* ^*flox*/*flox*^ mice^36, 37^ with CD11c-Cre mice^44^ (abbreviated as KO mice hereafter). Despite the tremendous heterogeneity of DCs^45^, multiple studies including a recent one from Esterházy, *et al*.^2^ showed that CD11c-cre mediated recombination occur selectively in DCs. In some experiments, the KO mice and WT littermates were further crossed with CX_3_CR1-GFP reporter mice^46^ to define CX_3_CR1^+^ population by GFP expression (Fig. 1c–e). We found that KO mice were developmentally normal and fertile. Lineage analysis by flow cytometry showed no major defects in general hematopoiesis including the development of T cells, B cells, macrophages, NK cells and granulocytes in the spleen (data not shown). Consistent with gp96 chaperone function, deletion of gp96 from CD11c^+^ cells resulted in decreased surface expression of CD11c, a known gp96 client (Fig. 1a, left panel). Due to CD11c expression level was decreased on gp96-deficient DCs, we used negative gating strategy (B220^−^MHCII^+^) to define DC lineages (Fig. 1a, right panel). The expression level of gp96 in various cells from WT and KO mice was compared. By intracellular staining, we found that the loss of gp96 was most in CD11c^+^ myeloid cells (B220^−^MHCII^+^), the majority of this population is DCs^47^. Consistent with the specificity of CD11cCre, gp96 expression was unaltered in CD11b^+^, Gr-1^+^CD11b^+^, B, T, and NK cells (Fig. 1b). We have thus successfully generated conditional gp96 KO mice with selective deletion of gp96 in DCs. To further determine the role of gp96 in DC function, we first checked surface expression of CD80, CD86, and ICOSL, which are important for providing costimulatory signals for T cell activation and survival. We found that the expression levels of CD80, CD86, and ICOSL are comparable between WT and KO DCs from spleen and mesenteric lymph node, except CD80 expression is decreased in KO splenic DCs (Fig. 2a–c). gp96 is also an essential chaperone for multiple integrins^36–38, 48^. To determine whether deletion of gp96 affects DC migration, we isolated DCs from the spleen and performed migration experiment *in vitro*. We found that the migration ability of DCs from KO mice was reduced in response to the CCL21 (Fig. 2d). Furthermore, we tested whether KO DCs had defects in antigen uptake and processing using fluorescence labeled chicken ovalbumin (OVA). Although the KO DCs had slightly decreased antigen uptake (Fig. 2e), we found that they processed OVA protein equally well comparing with WT DCs (Fig. 2f). We also generated bone marrow-derived DCs from the WT and KO mice and found that these KO cells failed to respond to stimulation by TLR2, TLR4 and TLR9 ligands, consistent with the need for gp96 in TLR folding (Fig. 2g). These data suggest that gp96 indeed controls multiple aspects of DC function and selective ablation of gp96 renders DCs defective in response to microbial cues without affecting their survival.

We are particularly interested in the function of CD11c^+^ cells at the mucosal surface. Among many heterogeneous DC populations in the intestine, two major subsets in the lamina propria have been identified based on expression of CD103 and CX_3_CR1^5, 6^. To examine if our KO mice have alterations of these two subsets, we isolated immune cells from mesenteric lymph node (MLN) and cLP from both KO mice and their WT littermates, and performed comparative analysis. The flow analysis of DCs was based on gating of MHC class II high population without B cell marker, i.e., B220^−^MHCII^+^ cells which can be further separated into CD103^+^, CX_3_CR1^+^, CD103^+^CD11b^+^, and CD103^+^CD11b^−^ subpopulations (Fig. 1c). We found that there was a dramatic increase in CX_3_CR1^+^ DCs and a reduction in CD103^+^ DC subsets in the KO mice compared to the WT littermates in both mesenteric lymph nodes (MLN) (Fig. 1d) and cLP (Fig. 1e), suggesting that the loss of gp96 from CD11c^+^ cells may tip the balance of DCs from tolerogenic to inflammatory ones.

### Deletion of gp96 from CD11c^+^ cells results in alteration of T cell immunity in the colonic lamina propria

**Figure 3:**
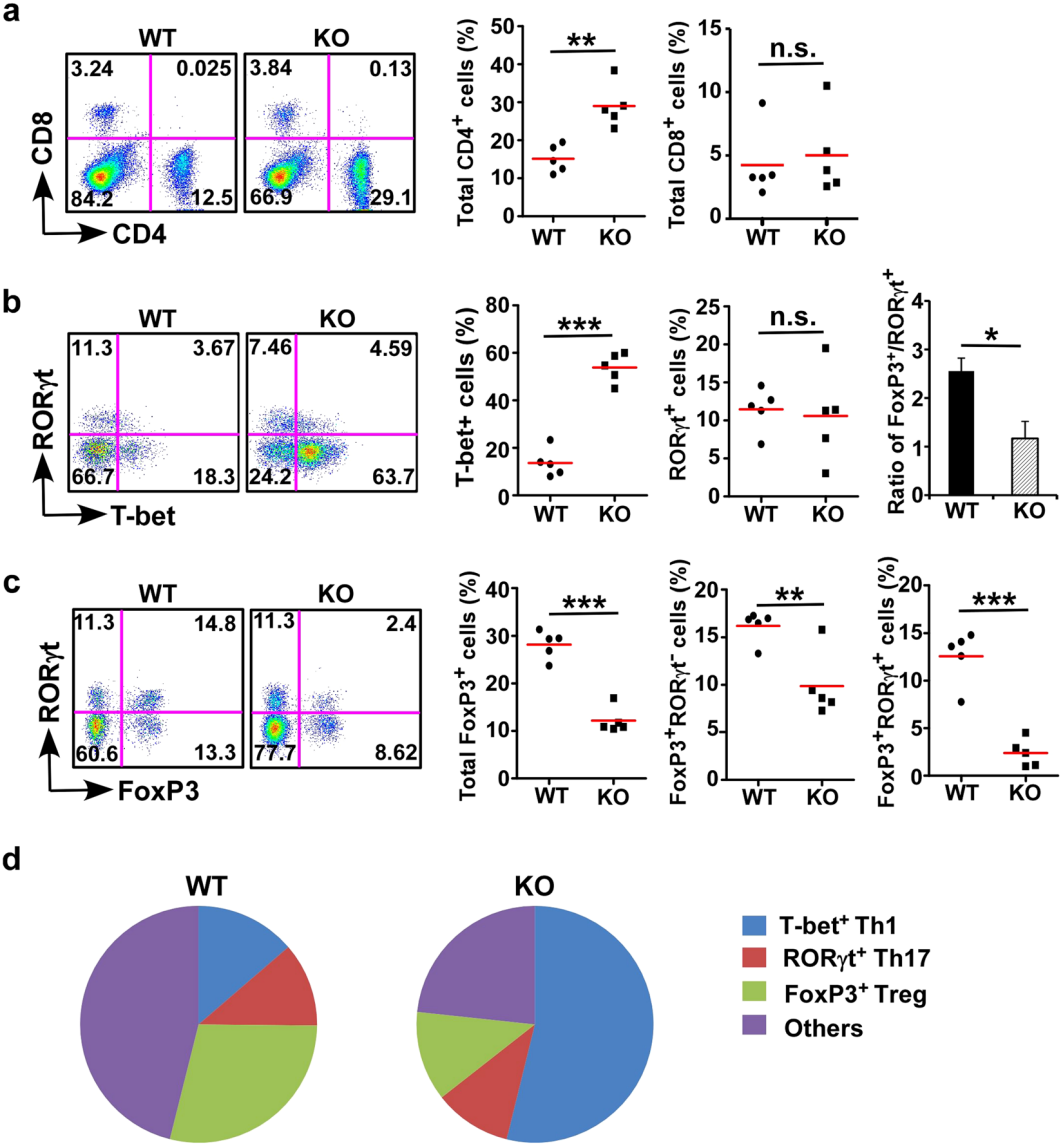
Deletion of gp96 in CD11c^+^ cells results in alteration of T cell immunity in cLP. (**a**) A representative flow cytometry analysis of CD4 and CD8 T cells (left) and quantification of CD4^+^ and CD8^+^ T cells in cLP of WT and KO mice (right). (**b**) Flow cytometry analysis of different subsets of CD4^+^ T cells (left), quantification of different CD4^+^ T cell subsets in cLP of WT and KO mice (middle), and the ratio of FoxP3^+^/RORγt^+^ subsets (right). (**c**) Flow cytometry analysis of FoxP3^+^ Treg cells (left) and quantification of different FoxP3^+^ Treg cells in cLP of WT and KO mice (right). Analysis of different CD4 subsets was based on positive gating of CD4^+^ CD3^+^ population. Numbers represent % of cells in each quadrant. Five mice per group were used. **p < 0.01; ***p < 0.001; n.s.: not significant. Data are representative of three independent experiments. (**d**) Proportion (average percentage) of CD4^+^ T cell subsets, among CD3^+^CD4^+^ cells.

In the intestine, DCs and macrophages play an important role in orchestrating CD4^+^ T cell responses. CD103^+^ LP DCs preferentially promote the differentiation of Treg cells^12, 13^, whereas CX_3_CR1^+^ LP DCs and microphages can induce Th1 and Th17 development in the gut. To determine whether there were altered mucosal CD4^+^ T cell subsets upon deletion of gp96 from CD11c^+^ APCs, we isolated cLP from KO mice and WT littermates, followed by examination of T cell subsets. We found that KO mice have more CD4^+^ T cells in cLP than WT littermates, whereas the percentage of CD8^+^ T cells appears comparable (Fig. 3a). Given the critical roles of the Treg-Th17 rheostat in immune tolerance, we further examined various CD4^+^ T cell subsets by intracellular staining of lineage-specific master transcription factors. Interestingly, we found that T-bet^+^ T cells (Th1) were significantly increased in KO mice compared with WT littermates, while RORγt^+^ T cells (Th17) remained the same between WT littermates and KO mice (Fig. 3b). Strikingly, the KO mice have significantly decreased FoxP3^+^ Treg cells in the gut, including both natural FoxP3^+^RORγt^−^ Treg cells and inducible FoxP3^+^RORγt^+^ Treg cells (Fig. 3c). Also, the ratio of FoxP3^+^ v.s. RORγt^+^ cells was decreased in KO mice (Fig. 3b, right). Taken together, we demonstrate that the mice with deletion of gp96 in CD11c^+^ pAPCs have altered CD4^+^ T cell distribution, with reduction of Treg cells and corresponding increase of inflammatory Th1 cells, underscoring the importance of gp96 in DC biology (Fig. 3d).

### gp96 in CD11c^+^ cells is required for inducing antigen-specific Treg cells

**Figure 4:**
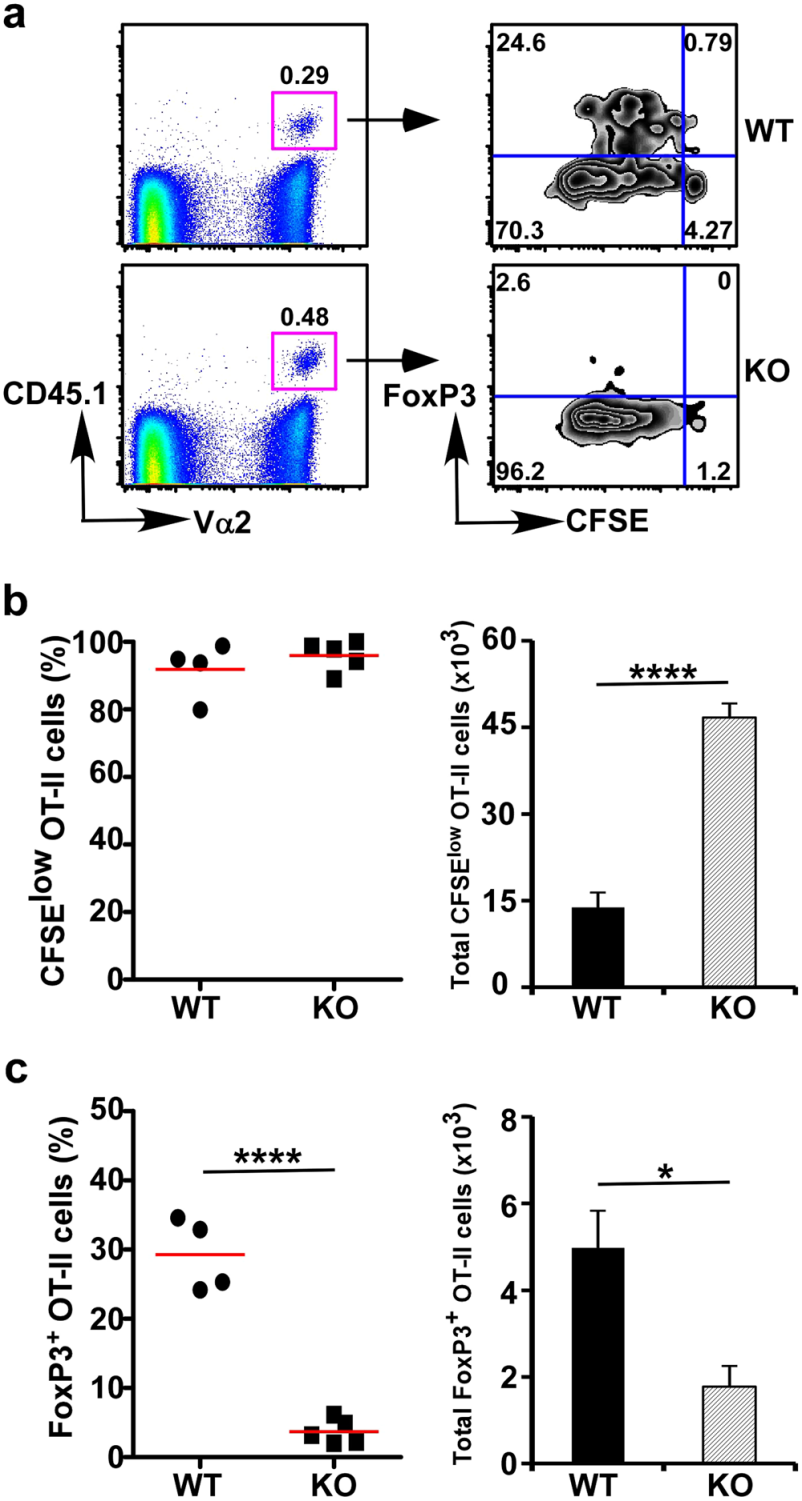
gp96 in CD11c^+^ cells is essential for inducing antigen-specific CD4^+^ Treg cells. (**a**) A representative flow cytometry analysis of naïve CD4^+^ OT-II donor T cells for proliferation by CFSE dilution assay and FoxP3^+^ Treg cell differentiation from naïve CD4^+^ OT-II T cells in MLN. (**b**) Quantification of CD4^+^ OT-II T cell proliferation (percentage of CFSE^low^ population, left, and total number of CFSE^low^ population, right). (**c**) Quantification of Foxp3^+^ Treg OT-II cells as shown in (**a**) (percentage of FoxP3^+^ OT-II T cells, left, and total number of FoxP3^+^ OT-II T cells, right). Each symbol represents one mouse. Error bars indicate SEM. *p < 0.05; ****p < 0.0001. Data are representative of three independent experiments.

Oral tolerance is a state of immunological nonresponse to dietary antigens and commensal bacteria^49^. A number of different mechanisms have been implicated in oral tolerance, in particular, the active induction of Treg cells^50^. Studies have shown that DCs are important for maintaining oral tolerance and preventing food allergies as well as inflammatory bowel diseases^51^. In addition, a recent study showed that CD103^+^CD11b^−^ DCs are required for peripheral Treg cell induction upon dietary antigen exposure^2^. Given the fact that deletion of gp96 from CD11c^+^ APCs results in alteration of CD103^+^ and CX_3_CR1^+^ subsets and decreased Treg cells in the lamina propria, we next examined whether gp96 in CD11c^+^ cells is required for inducing antigen-specific Treg cells. We induced oral tolerance against a model antigen: chicken ovalbumin (OVA). In brief, 8–12 week old KO mice and WT littermates were fed with 1% OVA in drinking water for 3 days, followed by adoptive transfer of 5 × 10^4^ CFSE labelled naïve OVA-specific CD4^+^ T cells isolated from OT-II T cell receptor transgenic mice. After four additional days of OVA feeding, these mice were scarified for examination of the priming and differentiation of donor-derived OT-II T cells in the MLN. We found that OT-II cells proliferated equally well in WT and KO mice as indicated by similar degrees of CFSE dilution (Fig. 4a and b), suggesting that gp96 in CD11c^+^ cells is dispensable for presentation of OVA. However, strikingly, OT-II CD4^+^ T cells efficiently differentiated into FoxP3^+^ Treg cells only in the WT mice (29.25 ± 5.26%) but not the KO recipients (3.37 ± 1.91%, p < 0.0001) (Fig. 4). Consistently, the total number of OT-II specific FoxP3^+^ cells in the WT mice (4.98 ± 0.86 × 10^3^) were significant higher than KO recipients (1.77 ± 0.48 × 10^3^, p < 0.05) (Fig. 4c, right). This data clearly indicated that gp96 is required for CD11c^+^ APCs to prime antigen-specific Treg cells in the gut.

### CD11c^+^ cell-specific gp96-deficient mice develop spontaneous colitis

**Figure 5:**
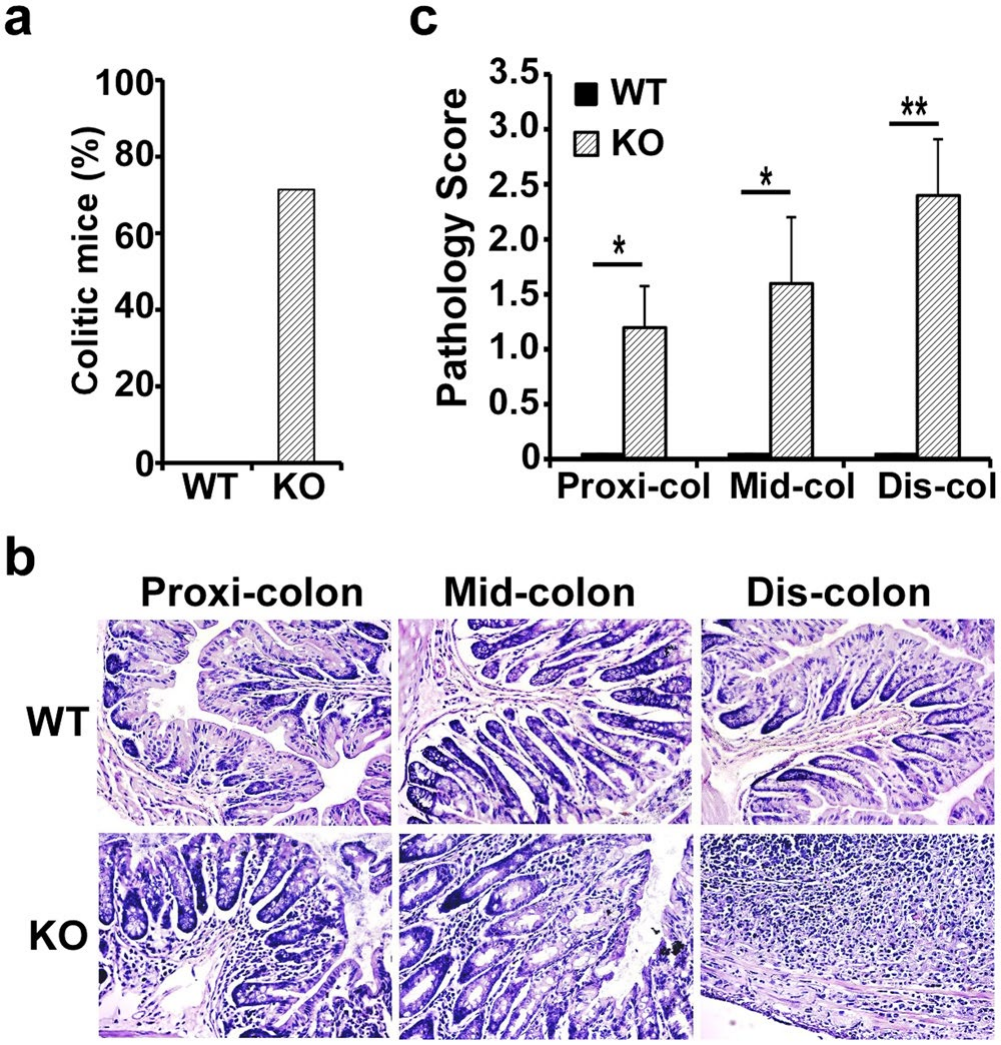
CD11c^+^ cell-specific gp96-deficient mice develop spontaneous colitis. (**a**) Colitis incidence of WT and KO mice at 24 weeks of age (n = 5 per group). (**b**) A representative image of H&E staining of colon cross sections from 24-week-old WT littermates and KO mice. (**c**) H&E staining in *B* was scored based on the pathology criteria detailed in Materials and Methods (n = 5 per group). Error bars indicate SEM. *p < 0.05; **p < 0.01.

Dysregulation of interactions between the gut microbiota and the mucosal immune system causes unchecked intestinal inflammation^31^. Given the fact that CD11c^+^ cell-specific gp96 KO mice have reduction of Treg cells in the gut and are unable to mount oral tolerance, we decided to closely monitor KO mice for signs of spontaneous intestinal inflammation and abnormalities. Young KO mice (6 to 12 weeks) did not display any apparent developmental defects. Intriguingly, at 24 weeks of age, 70% of KO mice developed spontaneous colitis (Fig. 5a). Histological evaluation and quantitative morphometric analysis of the colon revealed that KO mice developed severe inflammation with overt infiltration of mononuclear cells in the colon mucosa, focal ulceration, extensive mucosal damage, and loss of goblet cells (Fig. 5b). Based on several standard histopathological changes including degree of infiltration by neutrophils, mucosal damage and loss of goblet cells, we found clear evidence of moderate colitis in the entire colon of KO mice, but none in the WT mice (Fig. 5c). Thus, gp96 expression in CD11c^+^ APCs is essential to prevent dysregulation of the gut immune cells as well as the development of colonic inflammation. Our CD11c^+^ cell-gp96 KO mice thus represent one of the rare models of spontaneous colitis.

### CD11c^+^ cell–specific gp96 KO mice produce high levels of IgA

**Figure 6:**
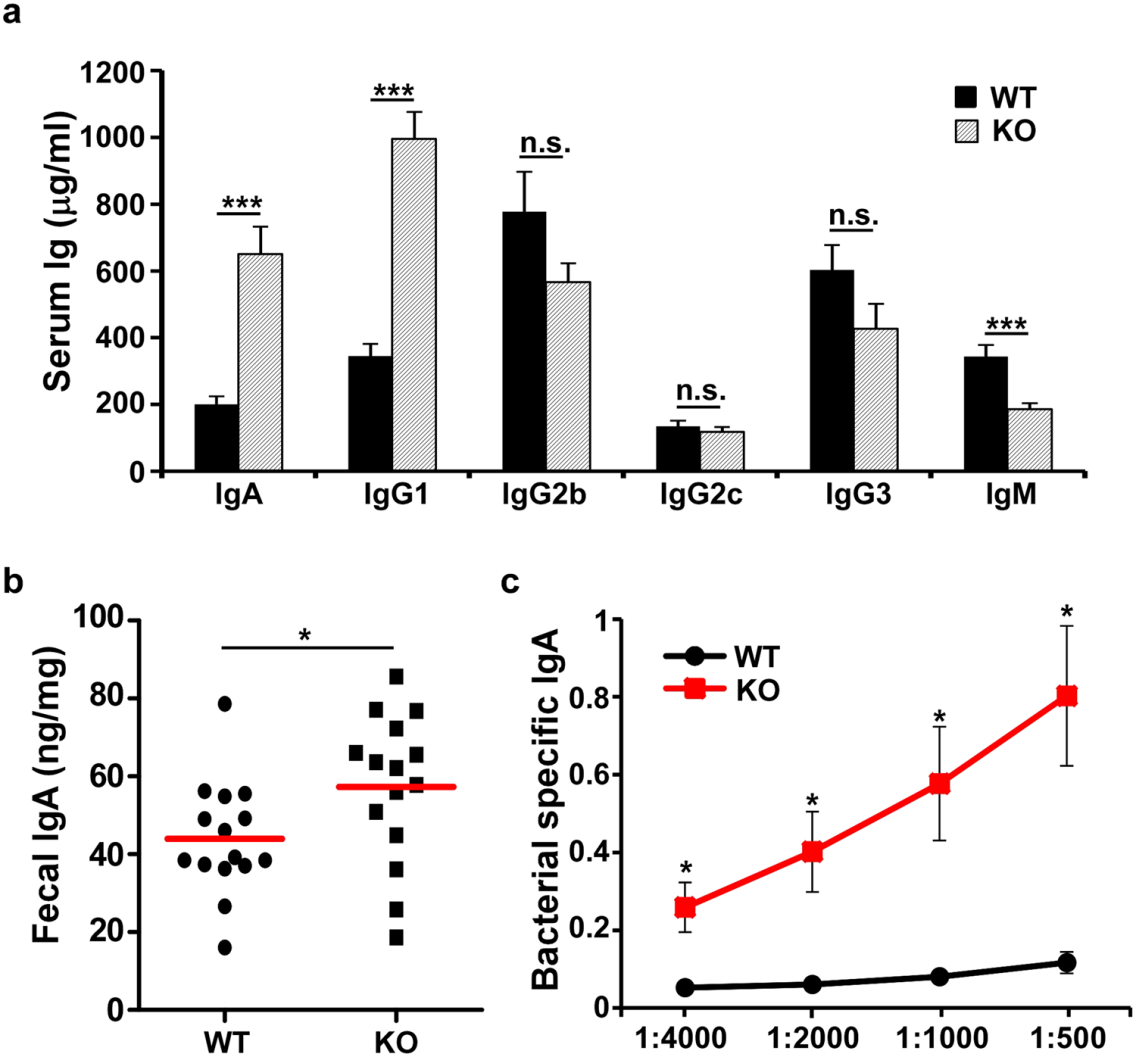
CD11c^+^ cell–specific gp96 KO mice produce high level of IgA. (**a**) Baseline Ig levels in the sera of 8- to 12-week-old WT littermates and KO mice were measured by ELISA (n = 7 per group). Error bars indicate standard error of mean. (**b**) IgA level in the fecal materials of 8- to 12-week-old WT littermates and KO mice were measured by ELISA (n = 15 per group). *p < 0.05; ***p < 0.001; n.s.: not significant. Data are representative of two independent experiments. (**c**) Bacterial specific IgA in the sera of KO mice and WT littermates were measured by ELISA (n = 5 per group). Error bars indicate SEM. *p < 0.05.

We so far demonstrated that CD11c^+^ cell-specific gp96-deficient mice developed spontaneous colitis with age (Fig. 5), as well as altered APC and T cell subsets (Figs 1 and 3). We next examined whether deletion of gp96 in CD11c^+^ APCs affects immunoglobulin (Ig) production and class switching to IgA, due to the important role of IgA in mucosal immunity and gut homeostasis. Indeed, intestinal DCs are required to regulate T cell differentiation for helping B cell differentiation^52^. It has been shown that CD103^+^CD11b^+^ DCs induce IgA class switching and generation of IgA-producing cells^53–55^. We measured systemic Ig levels at the steady state. We found that KO mice had significantly higher levels of IgA and IgG1 compared with WT controls at the baseline, while the levels of IgG2b, IgG2c, and IgG3 remained the same between WT littermates and KO mice (Fig. 6a). Moreover, the level of fecal IgA was significant higher in KO mice compared with WT littermates (Fig. 6b), consistent with the report that intestinal IgA coating represents ongoing inflammatory conditions^56^. As a further proof of loss of gut tolerance in CD11c^+^ cell-specific gp96-deficient mice, we measured the serum level of IgA against commensal bacteria. We found that the KO but not WT mice had dramatically increased bacteria-specific IgA (Fig. 6c).

### CD11c^+^ cell-selective gp96-deficient mice are highly susceptible to DSS induced colitis

**Figure 7:**
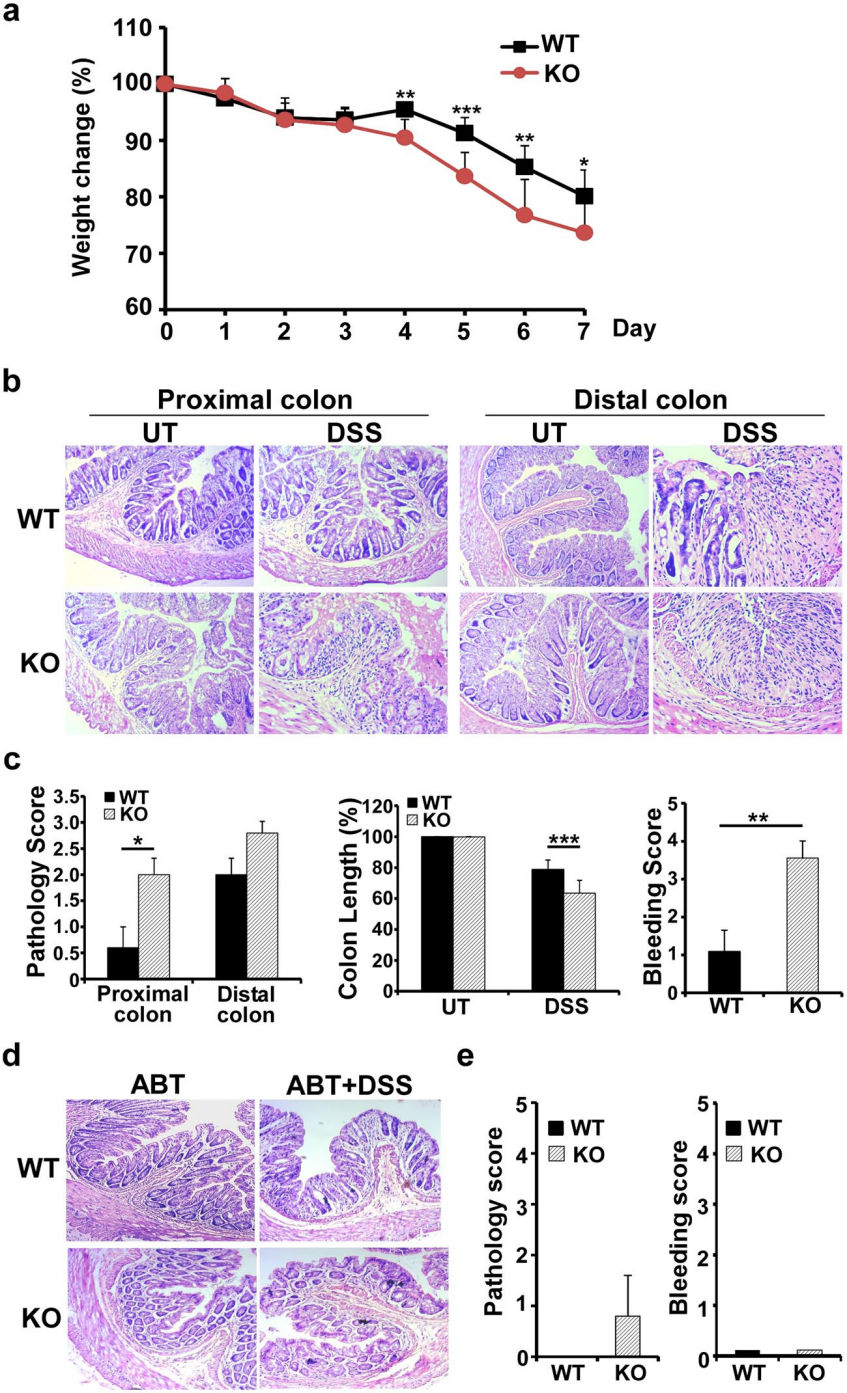
CD11c^+^ cell-specific gp96-deficient mice are highly susceptible to DSS induced colitis. (**a**) Body weight change of mice after DSS treatment. (**b**) A representative image of H&E staining of colon cross sections from untreated (UT) mice, or from DSS treated mice on day 7 (DSS). (**c**) H&E staining in (**a**) was scored based on the pathology criteria detailed in Materials and Methods (left); colon lengths were measured *ex vivo* (middle); and bleeding was scored on day 7 after DSS treatment (right). 8 to 12 week old mice were used (n = 5 per group). Error bars indicate standard deviation. *p < 0.05; **p < 0.01; ***p < 0.001. Data are representative of two independent experiments. (**d**,**e**) WT and CD11c^+^-specific gp96-deficient mice were treated with or without antibiotics for 4 weeks. Representative H&E staining of colon cross-sections (**d**) and the pathological score (**e**) were shown. n = 5 per group. Error bars indicate standard deviation.

Finally, we determined if the KO mice were more sensitive to DSS-induced colitis. WT and KO mice were given 5% DSS in the drinking water for five consecutive days. In agreement with the observation that KO mice were less tolerant to food antigens with spontaneous colitis, we observed that KO mice suffered more severe DSS-colitis (Fig. 7), with evidence of significantly more weight loss than WT littermates at day 7 (Fig. 7a). This was further corroborated by histological examination which revealed that colons of KO mice had higher amount of leukocyte infiltration with transmural inflammation, more extensive mucosal damage, increased loss of goblet cells, and ulceration (Fig. 7b). As a further indication of increased colitis, KO colons were significantly more shortened than WT colons. The gut of KO mice had severe damage, manifested by more hematochezia with higher bleeding score than WT littermates (Fig. 7c). To determine the effect of commensal bacteria in colitis, we treated WT and KO mice daily with a combination of broad-spectrum antibiotics (ampicillin, vancomycin, neomycin, and metronidazole) for 4 weeks. We found that removing commensal bacteria rescued both WT and gp96 KO mice from DSS-induced colitis (Fig. 7d), which indicated that dysbiosis plays an important role in our model. Collectively, we concluded that gp96 is required for the tolerogenic function of CD11c^+^ cells and gut homeostasis in mice.

## Discussion

The intestinal immune system is tightly regulated to maintain the balance between immunity and tolerance^57^. In the intestine, DCs and macrophages are strategically positioned to protect the gut integrity against pathogens while remaining tolerized to food, self-antigens and commensal microbes. The recognition of microbes by pAPCs is mediated by multiple pattern recognition receptors, including TLRs and NLRs^25–27^. We reported previously that heat shock protein gp96 is an essential immune chaperone for TLRs, integrins and other vital innate receptors^36–38^. In this study, we found that specific deletion of gp96 in CD11c^+^ cells altered both DC and CD4^+^ T cell subsets, favoring the proinflammatory CX_3_CR1^+^ DCs and Th1 cells over tolerogenic CD103^+^ DCs and Treg cells in the gut. The functional implication of our findings was evident from the development of spontaneous colitis in mice with selective deletion of gp96 in CD11c^+^ cells.

CD11c was initially discovered from intraepithelial lymphocytes. It was later found to be preferentially expressed by DC lineages in both human and mice. Despite occasional expression of CD11c by non-DCs, numerous fate mapping studies demonstrate that CD11c remains to be a reliable DC lineage marker, particularly in combination with MHC class II molecules to define DCs^47^. Not surprisingly, CD11c-cre mice have been used extensively in the literature to understand the developmental regulation of DCs^58, 59^, to address the function of DCs in innate immune defense^60^, priming T cells^61^, generating memory T cells in both physiological and pathological conditions^62^. More recently, by using this genetic approach, a study showed that DCs, but not macrophages is critical to establish tolerance in response to both Th1 and Th2 immunity^2^. Thus, using CD11c-cre mice, we have preferentially deleted gp96 from DCs. This was supported by no changes of gp96 level in other non-DC lineages such as T cells, B cells, NK cells, and macrophages. The loss of gp96 was restricted to Lin (CD4/CD8/B220/NK1.1/CD11b/Gr-1)^−^MHCII^+^ DC populations (Fig. 1).

We have previously generated several lineage-specific gp96 KO mice to delineate the contribution of gp96 and its clientele in host defense, including LysM-cre for macrophages^36^, CD19-cre for B cells^37^ and CD4-cre for T cells^41^. In particular, macrophage-specific gp96 KO mice were found to be resistant to DSS-induced colitis^43^. There was also no evidence of diseases in B cell-specific gp96 KO mice (data not shown). Thus, the central pAPCs in orchestrating gut tolerance appeared to be CD11c^+^ DCs. One overarching goal in the field is to define molecularly what dictates the tolerogenic program in DCs. Our current work illustrates that gp96 could be such an important molecule. As an essential chaperone for a myriad of innate receptors including almost all the TLRs, integrins, Wnt co-receptors and other yet unidentified molecules, gp96 can conceivably dictate the functional fate of DCs, analogous to the master transcriptional factor NFκB for regulating inflammation. Importantly, as a stress chaperone in the lumen of the endoplasmic reticulum, gp96 expression is up-regulated by unfold protein response (UPR) and other conditions that perturb the protein homeostasis. Thus, gp96 expression in DCs is likely to be tuned by both intrinsic and extrinsic factors such as microbiome and metabolic cues in regulating mucosal immunity. Given the important roles of gp96 in the biology of tolerogenic APCs as demonstrated in this study, the changes of the expression level of gp96 could thus have important consequences. Interestingly, a recent study indicated a strong association of loss of gp96 in gut APCs with Crohn’s disease^63^. Detailed phenotypic analysis of these cells shall be done to delineate the possible defect of gp96 expression by gut DCs in these patients. The recent understanding of the UPR sensor XBP-1s in inflammatory bowel diseases^64^ and cancer^19^ provide an intellectual precedent for our reasoning since gp96 also is a key downstream chaperone in the ER to mediate UPR.

Dysregulation of innate sensors somatically or in DCs usually do not cause spontaneous inflammation. For example, no colitis has been reported with TLR2-, TLR4-, TLR6- and MyD88-deficient mice^28–30, 65^. Although 35–40% TLR5 deficient mice do develop spontaneous colitis^66^, selective loss of TLR5 from DCs does not suffer from the same condition^67^. Our CD11c^+^ cell-specific gp96 KO mice develop spontaneous colitis at 24 weeks of age which are also highly susceptible to DSS induced colitis. Mechanistically, our data suggests the underlying alteration of T cell compartment is the major pathogenic factor. We found that deletion of gp96 from CD11c^+^ cells results in alteration of T cell subsets in the colonic lamina propria, with significantly increased T-bet^+^ T cells (Th1), and decreased FoxP3^+^RORγt^−^ and FoxP3^+^RORγt^+^ Treg cells compared with WT littermates (Fig. 3). Consistently, we further demonstrated that gp96 in CD11c^+^ cells is required for induction of FoxP3^+^ Treg cells in an oral tolerance model. We found that deletion of gp96 in CD11c^+^ cells did not affect OT-II cell proliferation in response to oral OVA in the MLN, suggesting that dietary antigen capture, processing and presentation to MHC class II to engage CD4 T cells are unaffected (Fig. 4), which is consistent with our *in vitro* data showed that loss of gp96 on CD11c^+^ cells did not affect antigen processing and only slightly decreasing antigen uptake (Fig. 2). However, KO mice failed to support the differentiation of naïve OT-II CD4^+^ T cells to FoxP3^+^ Treg cells (Fig. 4). Collectively, our work suggests a following instructive model for the roles of gp96 in CD11c^+^ cells in maintaining tolerance: step one: gp96 expression is required for differentiation of CD103^+^ tolerogenic DCs; step two: CD103^+^ tolerogenic DCs primes antigen-specific Treg cells via gp96-dependent manner; step three: Treg cells blunts the appearance of inflammatory Th1 cells in the gut; step four: gut tolerance is accomplished.

We have shown that gp96 KO DCs failed to respond to stimulation by TLR2, TLR4 and TLR9 ligands *in vitro* (Fig. 2), which suggests that complete ablation of gp96 renders DCs defective in response to microbial cues. However, we also found that KO mice developed spontaneous colitis (Fig. 5) and T-bet^+^ T cells (Th1) were significantly increased in KO mice in the gut (Fig. 3). It is well known that IL-12 is the major cytokine for Th1 cell differentiation^68, 69^. In response to inflammations, both monocyte-derived DCs and macrophages produce IL-12^70–72^. Potential source of IL-12 in our KO mice includes inflammatory monocytes, gut macrophages, dendritic cells that escaped cre-mediated deletion of gp96, and other cell types yet to be characterized. One future direction is to define the source of various inflammatory cytokines in our novel KO mouse model in an effort to fully elucidate the pathogenesis of colitis due to DC-selective ablation of gp96.

Finally, it is unclear if the loss of tolerance in CD11c-specific gp96 KO mice is due to abrogation of some important client proteins of gp96 such as CD103. This question is difficult to answer using the current system. Without gp96, CD103 is lost, rendering it difficult, if not impossible, to specifically isolate gp96 KO CD103^+^ DCs for functional study. Future study may need to dissect specific clientele of gp96 in DCs to complete the whole picture on the roles of gp96 in regulating tolerance *in vivo*.

In summary, our study for the first time demonstrated that CD11c^+^ cell-intrinsic gp96 is essential for maintaining gut tolerance and prevention of colitis. How gp96 in DCs regulates T cell differentiation and whether deletion of gp96 in DCs induces dysbiosis or inflammation-associated colon cancer need further investigation.

## Methods

### Mice

CD11c^+^ cell specific gp96-deficient mice (CD11cCre^+^ *Hsp90b1* ^*flox*/*flox*^) and control littermates (CD11cCre^−^ *Hsp90b1* ^*flox*/*flox*^) were generated by crossing our *Hsp90b1* ^flox/flox^ (gp96 is encoded by *Hsp90b1*) mice^36^ with CD11c-cre transgenic mice^59^. These gp96-deficient mice were further crossed with CX_3_CR1-GFP transgenic mice^46^ (Jackson laboratory) to define CX_3_CR1^+^ population. OT-II TCR transgenic mice were purchased from Jackson laboratory and bred onto the Ly5.1 background. All animal experimental protocols were approved by the Medical University of South Carolina Institutional Animal Care and Use Committee (IACUC). All methods were carried out in accordance with federal regulation as well as established institutional guidelines and regulations.

### Reagents

Antibodies used for flow cytometry were obtained from BD Biosciences (Mountain View, CA), eBioscience (San Diego, CA), and BioLegend (San Diego, CA). gp96 Ab was bought from Enzo Life Sciences, Inc (Farmingdale, NY). Dextran sulfate sodium (DSS) was purchased from MP Biomedicals, LLC (Santa Ana, CA). Percoll was obtained from GE Healthcare Life Sciences (Pittsburgh, PA). Dispase was purchased from Worthington (Lakewood, NJ). All other chemicals were obtained from Sigma-Aldrich (St Louis, MO) and Fisher Scientific (Pittsburgh, PA).

### Flow cytometry

Surface staining of cells and flow cytometry were done as described previously^36, 73^. To stain gp96 intracellularly, cells were fixed in 4% paraformaldehyde at room temperature and permeabilized with ice-cold methanol on ice for 10 minutes. Then the cells were blocked for 1 hour with FACS buffer containing 10% goat serum, followed by staining with anti-gp96 antibody, APC labeled anti-rat IgG antibody for 30 minutes at room temperature. Cells were acquired on FACSVerse (Becton Dickinson, Franklin Lakes, NJ) and results were analyzed with the FlowJo software (Tree Star, Ashland, OR).

### *In vitro* migration assay

MHC II^high^ DCs were isolated from the spleens of WT and KO mice using MACS magnetic beads from B220^−^ population. DCs were then seeded into the top chamber of a transwell insert. The media containing CCL21 (250 ng/ml) was placed in the bottom. After 4 hours, live cells in the bottom chamber were collected and counted via flow cytometry.

### *In vitro* antigen uptake and processing assay

DCs were incubated at 37 °C with OVA-Alexa 488 or DQ-OVA (Molecular Probes, Inc, Eugene, OR) for 10 or 90 min respectively. Live cells with OVA uptake or OVA processing were analyzed by flow cytometry. DCs incubated at 4 °C were used as a negative control.

### Enzyme-Linked Immunosorbent Assays (ELISA)

Ig levels in the serum and IgA level in the fecal materials were determined by a sandwich ELISA kit from Southern Biotechnology Associates (Birmingham, AL).

### Detection of bacterial specific IgA

The feces from μMT mice (B cell deficient mice) were resuspended in 0.3 ml carbonate-bicarbonate buffer (pH 9.5), then homogenized by a mini homogenizer for 1 min. The bacterial extract was harvested after centrifugation at 13,000 rpm for 10 min. The protein concentration in the bacterial extract was determined by Bradford Protein Assay. The bacterial extracts (0.5 μg/well) were then coated onto a 96 well plate for overnight at 4 °C. The plate was washed and blocked with 2%BSA/10%NGS in PBS for 2 hours at room temperature. The serum samples from WT and KO mice were serially diluted in 1%BSA/1%NGS in PBS (1:500, 1:1000, 1:2000, and 1:4000) and added onto the plate. After incubation for 2 hours at room temperature, the plate was washed. The presence of IgA was detected by the standard procedure of ELISA.

### Histology

Tissue was fixed in 4% formalin at least overnight, and then switched to 30% sucrose-PBS overnight. Tissue was then frozen in OCT medium and kept at −80 °C. Five micrometers of sections were cut on a Shandon Cryotome and mounted on charged slides (Fisher Scientific, Pittsburgh, PA). Slides were processed for hematoxylin and eosin (H&E) staining by standard methods and examined by a light microscopy. Colitis pathology score was obtained as follows: 0, normal epithelium; 1, low level of (occasional) leukocyte infiltration, no structural changes; 2, moderate leukocyte infiltration in lamina propria, surface epithelial lesion, no ulceration; 3, high leukocyte infiltration with inflammatory cells extending into the submucosa, mucosal erosion, focal ulceration, moderate thickening of the colon wall; and 4, very high leukocyte infiltration with transmural inflammation, extensive mucosal damage, loss of goblet cells, high vascular density, thickening of the colon wall, ulceration.

### Dextran sulfate sodium (DSS) induced colitis

Mice were treated with or without antibiotics (1 g/L ampicillin, 500 mg/L vancomycin, 1 g/L neomycin, and 1 g/L metronidazole in drinking water) for 4 weeks. They were administered with 5% DSS dissolved in water and fed ad libitum for 5 days. Fresh DSS was provided on day 3. The mice were given regular drinking water from day 5 until day 7, at which point they were euthanized. Five mice per group were used. For antibiotics-treated group, antibiotics were continued during DSS treatment. Mice were weighed, and their stool was scored daily. Bleeding was scored as follows: 0, no bleeding; 2, occult blood-positive (color changes to green or blue); 4, gross bleeding. Colon length was measured *ex vivo* from proximal colon to rectum. Colitis pathology score was assigned by a board-certified gastrointestinal pathologist.

### Colonic lamina propria cell isolation

Large intestines were removed and flushed with PBS to remove luminal contents. The intestine was cut open longitudinally, and then cut into 2 cm pieces. Samples were transferred to 1 mmol/L dithiothreitol (DTT)-containing PBS and shaken for 10 minutes at room temperature. Then the samples were transferred to PBS with 5 mM EDTA and 10 mM Hepes and shaken at 37 °C for additional 10 minutes. Samples were minced and then incubated for 30 min at 37 °C with collagenase D (1 mg/ml; Roche), dispase (0.05 U/ml; Worthington) and DNase I (100 μg/ml; Sigma). Lymphocytes were collected at the interface of a 40%/80% Percoll gradient (GE Healthcare) and washed in RPMI. Live cells were counted using a hemacytometer after Trypan blue exclusion.

### The induction of antigen-specific CD4^+^ Treg cells *in vivo*

WT and KO mice were given 1% chicken ovalbumin (OVA) in the drinking water for 3 days, followed by adoptive transfer intravenously of 5 × 10^4^ CFSE labeled CD4^+^CD25^−^ OT-II cells. Then the mice were given 1% OVA in the drinking water for an additional 4 days. On day 7, the mice were euthanized, and the proliferation and T regulatory cell differentiation of OT-II cells (FoxP3 expression on CD4^+^ T cells gated on congenic marker CD45.1 and specific TCR Vα2) in the mesenteric lymph nodes were measured by flow cytometry.

### Statistical analysis

Error bars represent the standard deviation (SD) or the standard error of the arithmetic mean (SEM). Two-tailed *t*-test or ANOVA were used to compare variables between different groups. All statistical analyses were performed using Prism 5 software. Values of *P* less than 0.05 were considered to represent statistically significant difference.
